# Early Prediction of Hemodynamic Shock in Pediatric Intensive Care Units With Deep Learning on Thermal Videos

**DOI:** 10.3389/fphys.2022.862411

**Published:** 2022-07-11

**Authors:** Vanshika Vats, Aditya Nagori, Pradeep Singh, Raman Dutt, Harsh Bandhey, Mahika Wason, Rakesh Lodha, Tavpritesh Sethi

**Affiliations:** ^1^ Indraprastha Institute of Information Technology, Delhi, India; ^2^ CSIR-Institute of Genomics and Integrative Biology, New Delhi, India; ^3^ Academy of Scientific and Innovative Research (AcSIR), Ghaziabad, India; ^4^ Computer Science and Engineering, Shiv Nadar University, Greater Noida, India; ^5^ Department of Pediatrics, All India Institute of Medical Sciences, New Delhi, India

**Keywords:** hemodynamic shock, deep learning, ICU—intensive care unit, artificial intelligence, thermal imaging, computer vision

## Abstract

Shock is one of the major killers in intensive care units, and early interventions can potentially reverse it. In this study, we advance a noncontact thermal imaging modality for continuous monitoring of hemodynamic shock working on 1,03,936 frames from 406 videos recorded longitudinally upon 22 pediatric patients. Deep learning was used to preprocess and extract the Center-to-Peripheral Difference (CPD) in temperature values from the videos. This time-series data along with the heart rate was finally analyzed using Long-Short Term Memory models to predict the shock status up to the next 6 h. Our models achieved the best area under the receiver operating characteristic curve of 0.81 ± 0.06 and area under the precision-recall curve of 0.78 ± 0.05 at 5 h, providing sufficient time to stabilize the patient. Our approach, thus, provides a reliable shock prediction using an automated decision pipeline that can provide better care and save lives.

## 1 Introduction

Hemodynamic shock is an acute and initially reversible condition that can act as an early signal to future-end organ failure (Herget-Rosenthal et al., 2018). It manifests due to inadequate blood perfusion than what is essentially required. Such a condition could cause tissue malfunction leading to rapid organ deterioration which can eventually result in death. The mortality rate due to delay in detection and treatment of such shock is as high as 34% in ICU patients admitted in developing countries (Divatia et al., 2016). Thus, proactive monitoring and therapy of hemodynamic shock can prevent the risk of impending organ failure and mortality (Rivers et al., 2001; Early Goal-Directed Therapy Collaborative Group of Zhejiang Province, 2010; Vincent and De Backer, 2013). However, delayed or insufficient monitoring can increase the risk of hemodynamic shock. Modern ICUs are equipped with a large number of sensors that generate humongous amounts of data besides a large number of clinical investigations. ICU clinicians and nursing staff are exposed to this large quantity of data on a real time basis. The overall burnout can go as high as 80.5% which can contribute to delayed or insufficient care (Rotenstein, 2018). Moreover, the resource-limiting areas have a higher risk of shock ignorance mainly due to a lack of technological advancement and a low doctor-to-patient ratio (Bagcchi, 2015). Most of the methods dealing with the shock in current times are invasive or require repeated contact, making the patient prone to hospital-acquired infections. The noninvasive ways such as Noninvasive Blood Pressure monitoring and Ultrasonography are not continuous but require contact with the delicate skin of infants which are infection-prone too (Oranges et al., 2015). Techniques such as Electrocardiography (ECG) and photoplethysmography (PPG) (Liu et al., 2020; Nachman et al., 2020) can also provide cardiovascular parameters such as the respiratory rate and heart rate in a noninvasive and wireless way to serve as important factors for shock monitoring. However, their contact-based approach can prove to be distressing and epidermal damaging for the fragile skin of neonatal patients (Barbosa Pereira et al., 2018). Thus, the use of artificial intelligence (AI) and frugal open-source technologies such as low-cost noncontact thermal monitoring can be effective for shock management.

We have created a body thermal imaging data cohort for the pediatric population under the Safe ICU initiative which warehouses 1.5 million hours of physiological vital data, laboratory investigation, treatment charts, doctors, and nurse investigation charts (Sethi et al., 2017). We have used these data to build automated hemodynamic monitoring and prediction of the future onset of the hemodynamic shock pipeline. Nowadays, nonintrusive vital monitoring measures have been gaining popularity because of their feasibility. Measures such as quantified capillary refill time (Q-CRT) and peripheral perfusion index (PPI) are being used for hemodynamic monitoring as noninvasive and transmitted light-based methods for assessing peripheral perfusion (Falotico et al., 2020). Vitals such as respiration and heartbeat have been known to be measured noninvasively using a twin-core fiber (TCF)-based sensor (Tan et al., 2019). Some studies have been successful in tracking peripheral blood oxygen saturation (SpO_2_) using broad-band lighting and an RGB camera (Guazzi et al., 2015). Balakrishnan et al. (2013) take just an RGB video as input and are able to detect heart rate and beat lengths by monitoring the subtle head movements. Heart rate can also be measured through videos using DeepPhys (Chen and McDuff, 2018) and Eulerian video magnification (EVM) combined with wavelet transformation (Froesel et al., 2020).

Thermal imaging, another nonintrusive and noncontact modality, is now increasingly being used in medical literature to extract the temperature information from the body and used for studies such as breast cancer detection (Ng, 2009; Mambou et al., 2018) and even diabetes mellitus (type 1) detection (Selvarani and Suresh, 2019). Thermography of the skin surfaces to study blood flow, cardiac pulse, and breath rate (Pavlidis et al., 2007) is being used as a basis for broader works such as preliminary hypertension assessment (Thiruvengadam and Mariamichael, 2018). Thermography of the human hand and foot skin surfaces has also been used to study blood flow using spectral filtering (Sagaidachnyi et al., 2017). A similar use of the thermography sensor has been proposed to make the disease activity detection in rheumatoid arthritis patients (Pauk et al., 2019). Lately, thermal imaging has also been used to detect the respiratory rate of humans (Liu et al., 2019). For instance, in cardiorespiratory signal monitoring, Barbosa Pereira et al. (2018) use thermal fluctuations beneath the nostrils for measuring the respiratory cycle. Since human bodies can be considered black bodies with an emissivity value of the skin close to 0.98 (Bagavathiappan et al., 2009), the infrared energy, and hence temperature information, can be suitably tapped by IR detectors such as Thermovision-550 (Thiruvengadam and Mariamichael, 2018), MWIR-Pheonix-FLIR (FLIR Systems, 2016), and SeekThermal^®^(Seek Thermal, 2022) easily available in the market. Even though some of the aforementioned techniques might incur a higher cost due to the nature of the thermal imaging sensors used, our study uses a simple SeekThermal smartphone camera attachment which can feasibly be used to capture the thermal context of the body. During hemodynamic instability, redistribution of blood occurs which causes a temperature difference in the center and periphery of the body, which acts as one of the first possible indicators of the advent of shock (Lima and Bakker, 2005; Lima et al., 2009; van Genderen et al., 2012; Ortiz-Dosal et al., 2014). Our study leverages this temperature difference, that is, Centre-to-Peripheral Difference (CPD) (Bourcier et al., 2016; Houwink et al., 2016), to study hemodynamic shock with the abdomen as center and feet as peripheral regions (Lima and Bakker, 2005; Hasanin et al., 2017). The difference observed is more in the case of a shock than of no-shock (Lima and Bakker, 2005; Nagori et al., 2019). We chose SeekThermal^®^ Camera for our study as it has affordable, minimal hardware architecture and can be easily mounted on a smartphone. CPD has been exploited along with some vitals and the use of machine learning methods such as Histogram of Oriented Gradients features with Random Forest classifier for detection and prediction of shock with a single snapshot of an image at a time point (Nagori et al., 2019), but a single image snapshot would present only limited information as it may be an outlier caused by random fluctuation. This may reduce the robustness and reliability of the model output. In addition, using the handcrafted features for machine learning algorithms might cost us time. To expand upon the study, we aim to reduce the manual tasks performed to calculate the features using deep learning methods and work on the continuous time-series data, improving the efficacy of detection and prediction.

**FIGURE 1:**
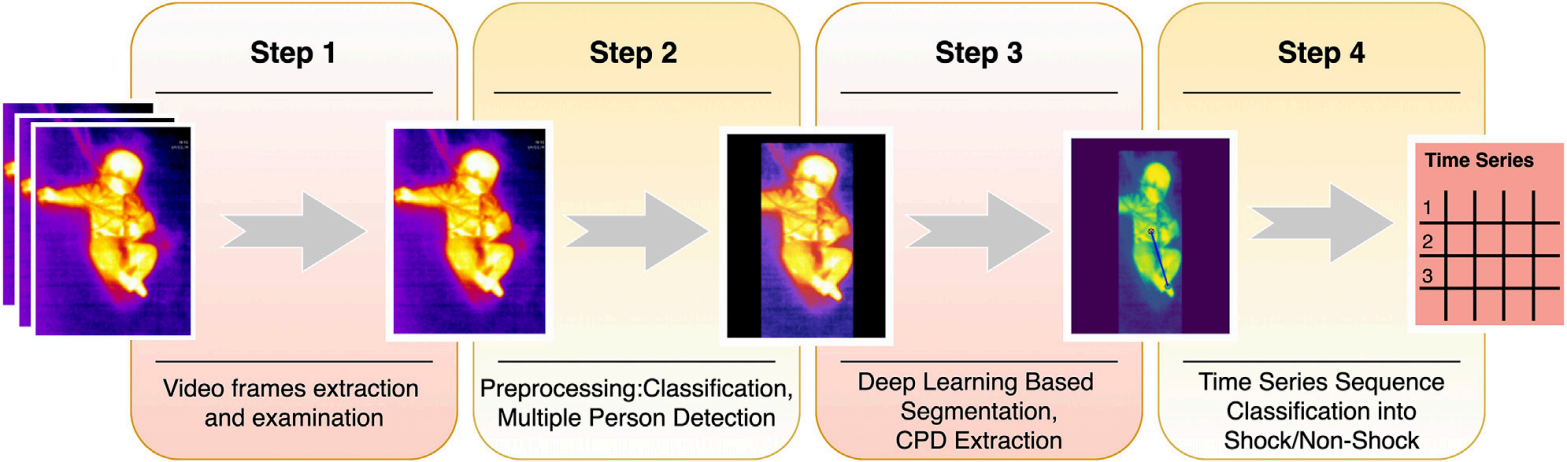
Shock prediction steps. The summary of the shock pipeline shows the steps from video frame extraction to shock prediction. Step 1 comprises sampling videos to extract frames. Step 2 classifies frames into covered or uncovered, while also finding the presence of multiple people in the frame and mask them, to avoid confusion. The masked images are then input to the ResUNet based segmentation model and CPD is hence extracted. The series of CPDs are then passed through a time-series sequence classifier, and finally, the predictions are made for shock for the next 6 h.

In this study, we developed a future risk prediction model (Figure 1) using continuously streamed videos of body infrared patterns and state-of-the-art computer vision (CV) and deep-learning (DL) techniques. Thus, the aim of our work was to build an automated, minimal contact pipeline for proactive hemodynamic monitoring. Automated detection and prediction will help prevent the adverse outcomes related to hemodynamic instability. Hence, the impact this problem creates and the scope of extending the solution make this study worthwhile in saving the lives of many pediatric patients.

## 2 Materials and Methods

For this study, we carried out thermal monitoring as a part of the SafeICU (Sethi et al., 2017) warehouse. We constructed an automated CPD extraction pipeline by body part segmentation on thermal videos using deep-learning models. Each time-point was labeled for shock and non-shock using an age-specific shock-index (Acker et al., 2017). The time-series data of the extracted CPD signals, combined with the heart rate and the labels, were preprocessed for case-control cohort design. We constructed cohorts at 0 h for detection and 1–6 h lead-time for prediction tasks. We then used multivariate Long Short Term Memory (LSTM) (Hochreiter and Schmidhuber, 1997) models to detect and predict the future onset of the shock. We finally evaluated the performance metrics of the models using a 10-fold cross-validation method.

### 2.1 Data Collection and Study Design

#### 2.1.1 SafeICU Framework

All the data reported in this research were collected from the Pediatric ICU of AIIMS, New Delhi, a tertiary care hospital in India. There were eight beds including neonatal beds in the pediatric ICU. We set up our servers to collect the real time physiological vitals periodic data. Vitals (heart rate, respiratory rate, and blood pressure) were routinely monitored using electronic sensors, that is, the heart rate was measured by ECG sensors, blood pressure was measured using arterial catheters, and respiratory rate and oxygen saturation were measured using pulse oximeter probes. Socket programming (network protocol) based on in-house software applications was used to extract the data from the Mindray™ central monitoring station (CMS). However, with an exception of heart rate, our study does not require these contact-based vitals for shock prediction, thus making the study of minimal contact. We also warehoused laboratory investigations, daily doctor and nurse notes, treatment charts, and thermal imaging.

#### 2.1.2 Ethical Approval and Patient Consent

The study was carried out with the approval of the Pediatric Intensive Care Unit of All India Institute of Medical Sciences, New Delhi, India. Since thermal images only capture infrared radiation, these did not reveal the patient’s identity and the study did not involve any contact or change in routine patient care. Hence, a waiver of consent was sought and granted by the Institute Ethics Committee (Ref. No. IEC/NP-211/08.05.2015, AA-2/09.02.2017). All experiments were performed in accordance with relevant guidelines and regulations as approved by the ethics committee.

#### 2.1.3 Cohort Based on Binary Shock Index

The SAFE-ICU described earlier has warehoused over 1.5 million patient hours of the monitoring data from the PICU. It is used to extract the time-stamped vital data for the patients at 0–6 h heart rate and blood pressure recordings. The shock index (SI) was calculated as the ratio of the median heart rate and median arterial systolic blood pressure, considered the gold standard for BP measurement in critical care settings (Pittman et al., 2004; Lehman et al., 2013). SI was computed over the median of moving sequential windows of 30 data points at a resolution of 15 s. Shock-index pediatric age-adjusted (SIPA) is used to compute shock/no-shock age-specific binarized outcomes for each patient (Acker et al., 2017).

#### 2.1.4 Thermal Imaging

Seek Thermal Compact (UW-AAA) with a resolution of 480*640 was used to capture thermal videos at 15 fps on iron theme settings. The camera, having a thermal sensor of 206*156 pixels was kept at a median (IQR) distance of 2.0828 m (0.1524) for long beds and 1.524 m (0.2665) for small beds. The ICU room temperature was maintained at 25°C. Temperature calibration was performed by matching the imager temperature with other known sources. Temperature from the thermal camera was found to be accurate with temperature measurement from other sources such as temperature probes and thermometers. Standard thermal video capturing and operating procedures were followed in order to ensure minimal disturbances by the extraneous factors, say, patient positioning, device handling, etc. (Supplementary Methods S1). Thermal cameras only capture infrared radiation so as to make sure that study does not reveal the patient’s identity. The camera was placed properly and at a good distance from the patient so that there was neither direct contact involved nor any change in patient routine care. The thermal videos were captured in a standard color scale guaranteeing that the full body of infants was visible. Thermal videos of every single patient were collected at different time points on different days. Thus each patient possibly has different values for shock status, which in turn eliminates bias due to the patient’s propensity characteristics, say gender, age, etc. Thermal artifacts were a lesser concern as we were calculating the relative gradient of temperature instead of the absolute temperature. Vital data with respect to the time-stamp of the videos were extracted from the data warehouse at 15-s intervals (SAFE-ICU) (Sethi et al., 2017). A comparison was made between the shock and non-shock groups using either the Wilcoxon rank-sum test or two-tailed Student’s *t*-test after testing for normality by the D'Agostino-Pearson normality test using GraphPad Prism version 6.00, GraphPad Software, La Jolla California USA.

### 2.2 Classification Into Covered and Uncovered

**FIGURE 2:**
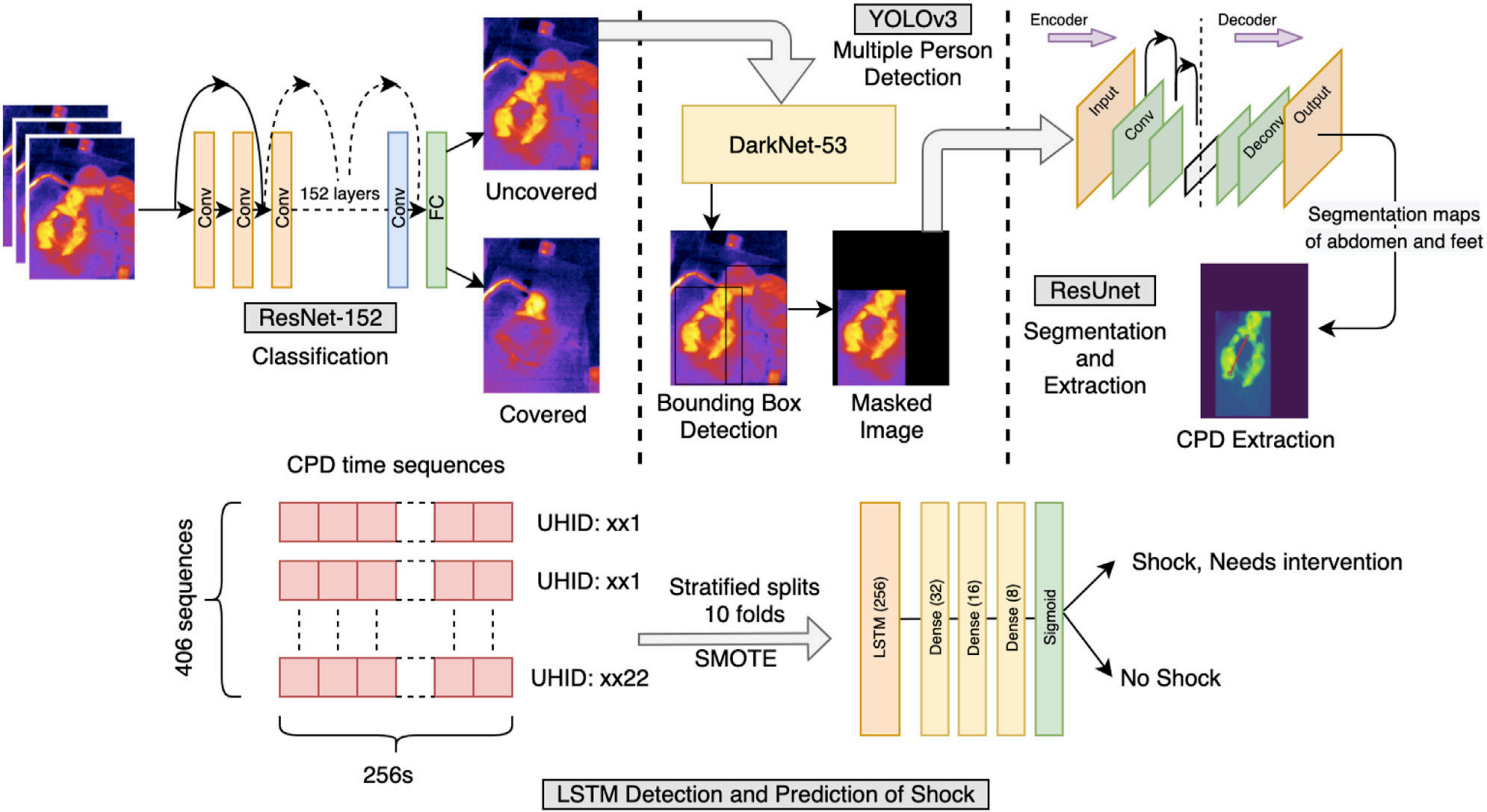
Shock prediction pipeline. Illustration of the pipeline followed for the detection and prediction of shock and no-shock. Each frame of the video was examined for uncovered/covered. The uncovered frames were then filtered off the presence of people other than the patient present in the frame. The frame was finally passed from the segmentation and CPD extraction model, further collecting the sequences used for LSTM time-series classification. The time sequences had CPD and heart rate as features and an appropriate window length of 256 s was chosen. Since the data were highly imbalanced, the SMOTE upsampling method was used in training the LSTM model. The detection and prediction of shock were carried out at 0 h and for the next 6 h, respectively.

The patients in ICU are kept under observation for a long duration. Being a very critical area, the patients are kept covered by a blanket most of the time, which is removed only for a short period, generally when a nurse/doctor comes to provide care. A ResNet-152 (He et al., 2016) architecture was trained using PyTorch (Paszke et al., 2019) framework in Python3 (Python Software Foundation, 2018) to classify each frame into covered and uncovered, i.e., abdomen and feet are visible (Figure 2). To train the data, images were augmented and normalized by their mean and variance especially extracted out to suit the thermal data. The model was finally implemented on videos sampled at 1 fps.

### 2.3 Multiple Person Detection

In the ICUs, caregivers providing care to the patient might come into the field of the camera mounted over the bed. For the CPD extraction task, bounding-box detection of multiple persons was used to filter out this additional presence so as not to confuse the algorithm. To avoid loss of crucial data where the caregiver and patient may be in the same frame, the bounding box corresponding to the larger area, that is, the patient was kept and the rest was masked. This region of interest was used for CPD extraction. For training purposes, a variety of images of the patient alone and along with the caretaker were manually annotated with bounding boxes using Yolo-Annotation-Tool-New (Murugavel, 2019) and augmented with linear transformations and Gaussian noise and passed through YOLOv3 (Redmon and Farhadi, 2018) based on Darknet-53 with a confidence threshold of 0.4.

The architecture had to be retrained from scratch as thermal data are very different from the COCO dataset, used in the original YOLOv3 manuscript. The YOLOv3’s output was the dimensions and position of the predicted bounding boxes detecting the people present in the frame, the correctness of which was verified through Intersection over Union (IoU) metric by calculating the ratio of overlapping of the predicted and ground truth bounding boxes with their union area.

### 2.4 Segmentation and CPD Extraction From Abdomen and Feet

Nagori A. et al. proved that the probability of shock depends directly on CPD. For this study, the abdomen was taken as the center and the peripheral was the foot. Images were annotated manually pixel-wise using js-annotator-tool. The target maps contained three one-hot encoded layers corresponding to the abdomen, feet, and background. The input images were normalized with mean and variance especially extracted from the distribution of the dataset in use. Appropriate image padding was carried out to ensure that the aspect ratio of images remains the same in case of any change in the input dimension. To account for a low dataset of pixel-wise segmented images for training, a ResUNet with ResNet-18, pretrained on ImageNet, was used as an encoder. UNet (Ronneberger et al., 2015) is specifically introduced to segment the less abundantly found medical data, helps to gather more local and global information even in the dearth of data, and thus efficiently segmenting out the images. A cutoff threshold was set on the predicted outputs to remove any weakly predicted pixels. The area detected was used as a region of interest in the original image and the mode of the detected probabilities was taken as the point of temperature extraction from the segmented-out abdomen and feet. The difference was divided by the abdomen value to keep CPD robust from thermal noise.

$$\text{Difference percent}=\frac{\left(AbdomenIntensity-FootIntensity\right)}{AbdomenIntensity}\times 100.$$

### 2.5 LSTM Time Series Sequence Classification

The videos were sampled at 1 fps to extract the CPD data from every uncovered window possible. Windows of 256 data-points corresponding to 256 s (4.26 min, padded, if necessary) were taken as an input to the LSTM-based classifier. The windows greater than 256 were split in an overlapping fashion. Each CPD, along with the heart rate at its corresponding time point was taken to finally label it with the shock index, and hence, the presence of shock/no-shock. The missing heart rate data at certain points were imputed with linear interpolation if the missing data were less than 10% of the time series length. Since the data are highly imbalanced with more non-shock sequences, the train-set is augmented with SMOTE (Chawla et al., 2002). The LSTM sequence classifier was followed by a series of dense layers with a dropout of 0.2, which then passed through a sigmoid layer to output binary shock index, and hence, shock/no-shock.

Experiments on tsfresh (Christ et al., 2016) feature classification with linear-mixed-effects models (Bates et al., 2015) and random-forests (Cutler et al., 2012), and the direct end-to-end classification of images/videos are shown in Supplementary Methods S2.

### 2.6 Outcome Variable—Binary Shock Index

The SAFE-ICU initiative has enabled this research to gather the PICU data and extract vitals and corresponding timestamps at the 0th hour (time of video capturing) and the next 6 h. The shock index was taken as the median heart rate and the median arterial systolic blood pressure, for moving sequential windows of 30 data points at a resolution of 15 s. SIPA (Acker et al., 2017) was then used to compute the age-specific binary outcome for each patient.

### 2.7 Model Evaluation

The video data were first partitioned patient-wise such as to keep train, validation, and test sets unseen from each other. For the 10-fold cross-validation, the data were partitioned with a ratio of 60:20:20 into these three sets in a stratified manner, that is, keeping the distribution of low-percentage shock class comparable in all three sets. Training data was augmented for the low-found shock class using SMOTE; the validation and test sets remain unchanged in their size in each respective fold. The model analysis was mostly conducted on the area under precision-recall curve (AUPRC) and the area under receiver operating characteristic (AUROC) curve. Other standard metrics like F1-score, PPV, NPV, specificity, and sensitivity were evaluated at the Youden’s Index (J) (Ruopp et al., 2008). Since there is a high significance of prevalence in the medical domain, calculating the metrics at the Youden’s Index becomes important.

## 3 Results

### 3.1 Patient Characteristics, Preprocessing, and Cohort Building

Statistical inferences of the cohort characteristics between shock and non-shock groups are depicted in Table 1. Shock and Non-shock conditions were derived using the age-specific shock index cutoff values. It can be observed that the most significant difference between the two categories is in the heart rate, as expected, along with the respiratory rate. We obtained a median length of stay of 6.808 days with a total of 132 shock instances and 274 non-shock instances. The study uses arterial systolic blood pressure, the gold standard for BP measurement in critical care settings (Pittman et al., 2004; Lehman et al., 2013), from 22 patients monitored longitudinally as a required vital for the calculation of shock index for the training purposes which is available for only a small subset of patients. However, once the model is trained, this is no longer a limitation at inference time as the trained model does not require blood pressure for inference (making a prediction of shock status). The pipeline uses >1,00,000 frames and the corresponding shock indices for training the prediction models.

**TABLE 1:** Cohort characteristics and statistical significance of control (non-shock) vs. affected (shock) classes. The *p*-values were calculated using either the Wilcoxon rank sum test (W) or Student’s t-test (t) after testing for normality by the D’Agostino-Pearson normality test. (*n*, number of sequences; IQR, interquartile range).

Variable	Non-shock seq (*n* = 274)	Shock seq (*n* = 132)	Statistical tests
	Median (IQR)	Median (IQR)	*p*-value (W/*t*)
Age (months)	60.12 (36.07)	75.89 (107.02)	0.6087 (W)
Arterial systolic blood pressure, mm Hg	131.94 (19.56)	128.09 (25.21)	0.0014 (*t*)
Systolic blood pressure, mm Hg	106.00 (20)	102.00 (5.00)	0.002 (W)
Heart rate, per min	106.16 (19.14)	143.17 (63.81)	**<0.0001 (*t*)**
Respiratory rate, per min	32.34 (13.14)	22.87 (13.20)	**<0.0001 (W)**
O_2_ saturation (SpO2)%	97.50 (2.68)	98.32 (3.45)	0.9932 (W)

The bold values symbolize statistically significant characteristics.

### 3.2 Segmentation of Abdomen and Feet Achieved a Total Dice Loss of 0.0391 Using ResUNet

Since thermal images lack texture, it is important for the model to recognize the structural shape features. ResUNet was specifically used to make the task possible on relatively less available thermal data and to capture both the local and global information from the image. A dice loss of 0.0391 (dice coefficient = 0.9609) with a Binary Cross-Entropy loss of 0.0692 was achieved for segmenting out the abdomen, feet, and background. The mode intensities of segmented areas were then used to find out CPD and hence build the longitudinal models from continuous long-duration videos. The results are showcased in Supplementary Table S1.

### 3.3 The LSTM Model Was Found to be the Best Performing

**FIGURE 3:**
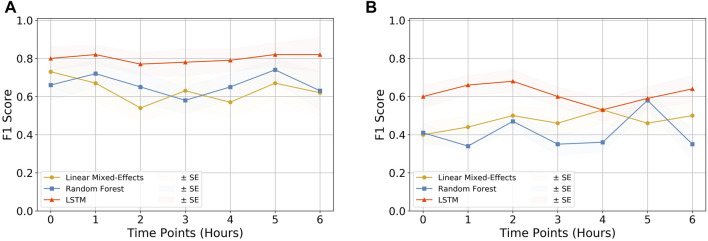
Quality assessment of models. The F1 score comparison for the three models (i.e., Linear Mixed-Effects model, Random Forest, and LSTM) tested on **(A)** CPD and heart rate and **(B)** CPD parameter only. It can be observed that the LSTM model outperforms the other two models in both cases, making it the primary choice of this research. The rest of the comparison plots are depicted in Supplementary Figure S1.

We compared three models to finally arrive at the best-performing one. Linear-Mixed Effects (Bates et al., 2015), Random Forest (Cutler et al., 2012), and LSTM (Hochreiter and Schmidhuber, 1997) were tested at various time points from the observation taken. Based on various metric evaluations, LSTM was found to be the best performing on our given time series data and hence was chosen as the primary choice for our study. The F1 score comparison of the three models is shown in Figure 3, and the rest of the evaluations are shown in Supplementary Figure S1.

### 3.4 Evaluation of the LSTM Model at Different Lead Times Reveals That CPD Increases the Model Performance

**FIGURE 4:**
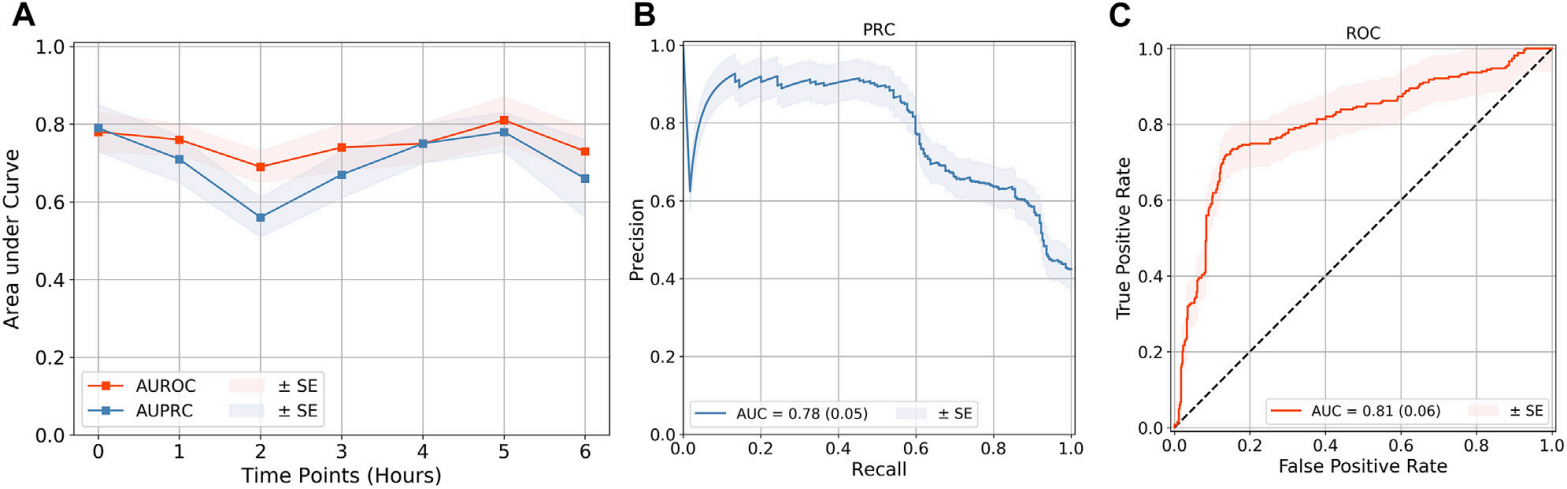
Quality evaluation on the LSTM model. **(A)** Quality evaluation of the LSTM time-series classification models at lead points of up to 6 h. The best performance of **(B)** AUPRC and **(C)** AUROC was obtained at a 5-h prediction. The rest of the performance metric results are demonstrated in Supplementary Figure S2. The results of all are shown in Table 2. The standard error (SE) for each is calculated from cross-validation, by taking *k* = 10.

The SAFE-ICU resources allowed us to match the time sequences with their corresponding states of shock/non-shock for the next 6 h since the observation was taken. We tested the LSTM model performance for 1–6 h of lead time on CPD and the heart rate as shown in Table 2. As shown in Figure 4, we got the best performance at AUROC 0.81 ± 0.06 and AUPRC 0.78 ± 0.05 at 5 h. This gives a good time window to alert the medical practitioners to start the therapy for preventing the advent of shock.

**TABLE 2:** Performance of the proposed model predicting the presence of shock/non-shock using automated CPD and heart rate. The time pt. column depicts the subsequent hours from the time of taking the observation, at which the results were recorded. The unequal number of shock and non-shock sequences is due to the absence of patient data with the increasing number of hours. (S/NS, number of shock/non-shock sequences present; AUPRC, area under precision-recall curve; AUROC, area under receiver operating characteristics; PPV, positive predictive value; NPV, negative predictive value; D, detection; P, prediction).

Time pt.	S, NS	AUPRC	AUROC	Accuracy	Sensitivity	Specificity	PPV	NPV	Youden
		Mean (SE)							Mean
0 h (D)	132, 274	0.79 (0.06)	0.78 (0.05)	0.85 (0.03)	0.74 (0.06)	0.92 (0.02)	0.86 (0.05)	0.84 (0.03)	0.50
1 h (P)	115, 271	0.71 (0.06)	0.76 (0.04)	0.83 (0.04)	0.83 (0.04)	0.82 (0.06)	0.80 (0.06)	0.88 (0.02)	0.42
2 h (P)	125, 253	0.56 (0.05)	0.69 (0.04)	0.83 (0.02)	0.72 (0.06)	0.90 (0.03)	0.82 (0.05)	0.84 (0.04)	0.56
3 h (P)	133, 232	0.67 (0.06)	0.74 (0.06)	0.83 (0.04)	0.69 (0.08)	0.93 (0.04)	0.88 (0.06)	0.82 (0.04)	0.57
4 h (P)	123, 242	0.75 (0.05)	0.75 (0.05)	0.81 (0.04)	0.70 (0.06)	0.94 (0.03)	0.90 (0.05)	0.78 (0.06)	0.64
5 h (P)	120, 247	0.78 (0.05)	0.81 (0.06)	0.84 (0.04)	0.76 (0.07)	0.94 (0.02)	0.88 (0.05)	0.84 (0.05)	0.62
6 h (P)	124, 228	0.66 (0.10)	0.73 (0.06)	0.89 (0.03)	0.82 (0.08)	0.92 (0.04)	0.81 (0.09)	0.95 (0.02)	0.62

## 4 Discussion

The study presents a computer vision and deep-learning-based continuous and noncontact shock detection and prediction model which leverages Center-to-Peripheral Difference (CPD) as one of its main parameters. Care needs to be taken to evaluate the parameters in a noninvasive way such as not to cause infections by contact. The role of noncontact, wireless health monitoring has gained much importance, especially during the times of the COVID-19 pandemic. The noncontact estimation of multiple physiological parameters can enable telediagnosis and remote management of patients, at the same time decreasing the contact-based infection risk. Among the available noncontact modalities, recent advancements in thermal imaging have made thermal imaging a key technology. Affordable smartphone thermal cameras have been used to detect the breathing rate and hence the perceived mental stress (Cho et al., 2019) and even help identify sports injuries (Quintana et al., 2015). In ICUs, studies providing noncontact measurements such as ECG (Majumder et al., 2018) and other physiological measurements using noncontact cameras (Jorge et al., 2022) have been explored. Wireless sensors have shown potential for the detection of complex underlying neurological disorders, such as Shah et al. (2019) use wireless sensor networks (WSN) for monitoring pill-rolling tremors which can be proven to be a precursor to Parkinson’s disease. Similarly, Liu et al. (2018) use WSN to detect and monitor abnormal breathing patterns as an underlying condition for more gruesome diseases like hyperthyreosis and sleep apnea. Since the field of thermal image inspection has only started to be explored, it can thus be leveraged, along with some noninvasively monitored vital parameters, for the prediction of shock. The use of deep learning methods proves to be really beneficial in reducing manual preprocessing and increasing the accuracy of methods.

In this study, we have extended our previous study on hemodynamic shock prediction with longitudinal continuous monitoring of body thermal patterns which are found to be predictive for future shock prediction (Nagori et al., 2019). Longitudinal monitoring of temperature gradient opens up a rich source of information about patient physiology. The underlying stochastic patterns can have a discriminative value for future hemodynamic shock risk. We leveraged these reasonings to extract the center-to-peripheral intensity difference from the thermal images in a time-series fashion. We do so by applying our data-specific trained screening filters for covered and uncovered patient detection models, and multiple person detection models, which are followed by segmenting the body parts into abdomen and foot using artificial intelligence–based models. The extracted CPD time series along with vitals were used to predict the future (1–6 h) hemodynamic shock using sequence models (LSTM). This model was also compared with Random-Forest and Linear Mixed-Effects models, known for working with longitudinal data but LSTM outperformed the other two. We compared the results of our method with Nagori A. et al.’s study too and observable improvement can be seen in the majority of the metrics (Supplementary Table S2). We also trained and checked the performance of models that can classify the length of videos and images for the future risk of hemodynamic shock. CNNs, which have shown state-of-the-art performance for image classification, were used for the task. However, we found that domain features, such as CPD, performed better than direct image and video classification for the future risk of shock. Even after many experiments performed for a direct classification of images using the concepts of TV-Chambolle (Condat, 2013) denoising, data augmentation, and undersampling/oversampling, we were able to get the best AUROC of only 0.60 for shock detection using ResNet-50. This can be because of the cluttered background with diapers and tubes, and an increased region of interest for the information extraction from a limited variety of images. This also tells the importance of the domain-specific features over end-to-end CNN-based approaches. We built the models for multiple time points till 6 h which showed the best performance at a lead time of 5 h. The metric AUPRC and F1 scores are most significant for an imbalanced dataset such as ours as it does not get biased by the presence of true negatives and thus gives a clearer perspective of a classifier’s utility. The results till 6 h show a promising window with a good prediction rate which can prove to be helpful for the doctors to help find a buffer time prior to the shock event and therefore start the treatment.

Our study currently applies to cold shock as neonates, and young children are more commonly present with “cold shock”—a state of elevated systemic vascular resistance (SVR) and low cardiac output with cold extremities due to vasoconstriction and delayed capillary refill (Martin and Weiss, 2015). The peripheral vasoconstriction can be identified with the center to peripheral temperature difference using thermal imaging that we leveraged in our study to identify the cold shock. We also acknowledge the limitations and uncertainty caused by using skin temperature and core-peripheral temperature gradient in isolation (Schey et al., 2010); therefore, we introduced the heart rate as a crucial cardiovascular parameter (Table 1) in our CPD-based method for shock prediction. Respiratory rate (RR) is also an important indicator of cardiorespiratory status (Liu et al., 2020) as indicated in Table 1. However, we did not encapsulate RR in our models owing to its limited availability at all-time points in our dataset and in a bid to keep the method parsimonious. Nevertheless, we did study the effects of including RR with the available measurements till 6 h, and it showed promising results as demonstrated in Supplementary Table S5, which can be taken up in our future research. Models that are predictive of future occurrence of events suffer from lower model performance indicators and a trade-off is needed between sensitivity and specificity. While probability thresholds can be easily set to decrease the false positives (at the cost of sensitivity), our model is intended to be used as a screening tool as a shock is a potentially lethal condition. Moreover, the FPR varies between 10 and 15% at different time points and certainly can be improved further with the incorporation of additional features. We will be taking up further performance improvement in our future research to bring it down to less than 5%. The other limitations of our method arise during the quality data procurement. Monitoring thermal patterns could be hard at times when the patient is being covered or being operated on; caregivers can also block the recordings. However, these issues can be resolved by keeping ∼4.3 min of uncovered slots for monitoring. Nevertheless, we have built an AI system that is efficient enough to identify the covered and uncovered images and can remove the caregiver or person other than the patient. Thus, our system is able to extract the thermal patterns efficiently. Using CPD as a co-acting parameter eliminates the use of invasive arterial monitoring and cuff-based blood pressure monitors which can cause infections due to the repeated use on multiple patients. Our models require thermal recordings which do not identify an individual’s identity, hence are safe for patient privacy and do not take much time away from the time assigned for care. Our methods can be extended beyond the intensive care settings, that is, to help in the community-based monitoring by the developing countries’ rural healthcare workers, such as the Accredited Social Health Activist (ASHA), run by the women promoting accessible healthcare in rural Indian settings. This can be made possible by training them to use a specifically designed frugal mobile app for thermal shock detection and prediction, which can work with readily available Android/iOS smartphones, with only model weights preinstalled. The results can be made more robust by expanding the dataset by including more patients in the study. Also, the dataset can be made varied and generalized by including observations from multiple other clinical sites. Thus, the study performed illustrates a great noninvasive and minimal feature architecture that promises to be a life-saver by informing the clinicians about shock well in advance.

## Data Availability

The raw data supporting the conclusion of this article will be made available by the authors, without undue reservation.
