# Supplementary material for: Early Prediction of Hemodynamic Shock in Pediatric Intensive Care Units With Deep Learning on Thermal Videos
Source: Front Physiol. 2022 Jul 11;13:862411. doi: 10.3389/fphys.2022.862411 (PMC9340772; doi:10.3389/fphys.2022.862411)
Supplement: Supplementary file 2 [file DataSheet1.DOCX]

**Supplementary Material**

**Supplementary Methods S1**

**Standard operating protocol (SOP) for capturing thermal videos**

1. Before entering the ICU, make sure that one has to wear shoe covers, a scrub, and a disposable gown.

2. Need to connect the Seek Thermal Camera to the available Smartphone and then open the Seek Thermal app.

3. Ensure that the Seek Thermal app is set to a high-resolution setting and to an “Iron Theme” before capturing videos. Wall mount hanger is used to hold a smartphone. Seek Thermal Camera is placed at a good distance from the bed and at a certain height in order to make sure that patients on the bed are not disturbed.

4. For each patient recorded in this session, the following steps were followed:

- Making the patient comfortable is the most important part. The camera was placed properly and at a good distance from the patient so that there was no direct contact involved nor any change in patient routine care.
- In the presence of a nurse, a smartphone is hung using a wall mount hanger.
- MSExcel sheets are used for data collection. Data such as UHID, bed number, date, and time of the patients on this session were recorded for identification purposes.
- After capturing videos, the mobile hanger is removed safely and the data is saved on a server, on a daily basis, into an identified folder with the patient’s UHID, bed information, and date.

**Supplementary Methods S2**

**Linear Mixed-Effects and Random Forest sequence classification on tsfresh features:** tsfresh features were extracted from 256-length sequences and trained on the same train and validation distributions as the previous LSTM model using ‘Boruta’ (Kursa and Rudnicki, 2010) package from R-language. Variation Inflation Factor (VIF) was used to reduce multicollinearity in data. If VIF exceeds 10, then the collinearity is considered problematic, and hence that particular variable causing it should be removed. The remaining features were used to train linear mixed-effects and randomforest models.

**Direct Classification of thermal images/videos for future risk of shock:** Apart from CPD extraction, an attempt was made to classify into shock/no-shock by directly giving whole images/videos as input. In one direction, we tried to classify each video frame read at a time and conducted experiments with several modern architectures based on convolutional neural networks (CNN). The concepts of TV-Chambolle denoising, data augmentation, and undersampling/oversampling, were used to get the best shock detection AUROC of 0.60 using ResNet-50 (Supplementary Table S3). Also, the information extracted from a single image frame can be very limited. So instead, we tried to use direct and continuous video samples of length 256s as an input to a conjunction of various CNN-LSTM models, trained in a time-distributed manner. Being a fundamental extension of the direct image classification problem, it suffered from similar limitations.

**Classification into covered or uncovered patients**

Since the patients are kept under observation for long hours in intensive care conditions, it is possible for them to remain wrapped in a blanket for most of the time. Using ResNet-152, we were able to classify the video frames into covered/uncovered to get a hold of any window during which the blanket is removed. An accuracy of 95.52% was achieved, with an F1 score of 0.9589 (Precision=0.9722, Recall=0.9459), creating fewer chances of false positives.

**Multiple person detection using YOLOv3**

The patient is barely left alone when he/she is uncovered under long hours of observations. To remove any chance of confusion between the temperatures and the body parts that the following segmentation algorithm might face, YOLOv3 was deployed for multiple person detection which achieved an IoU of 0.71206. The other person and the background were then masked to keep the patient in focus for CPD extraction.

**Supplementary Table S1.** Performance of models in the preprocessing steps in the tasks of classification, object detection and segmentation.

| Classification | | | |
| --- | --- | --- | --- |
| ResNet-152 | | | |
| Precision | Recall | F1-Score | Accuracy |
| 0.9722 | 0.9459 | 0.9589 | 0.9552 |

| Object Detection | Segmentation | | |
| --- | --- | --- | --- |
| YOLOv3 | ResUNet | | |
| IoU | BCE Loss | Dice Loss | Total Loss |
| 0.7121 | 0.0692 | 0.0391 | 0.0542 |

^
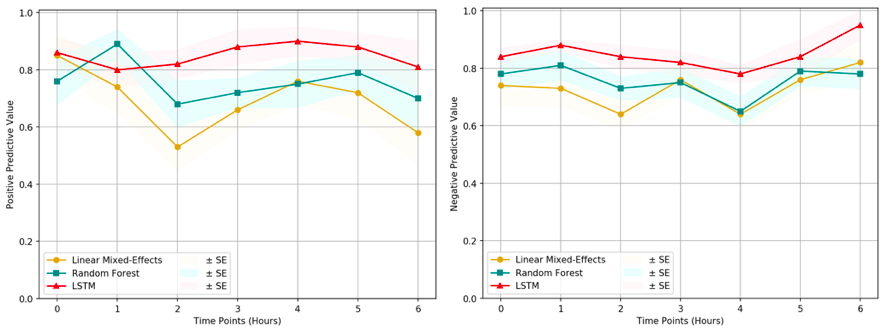
^

**A** **B**

^
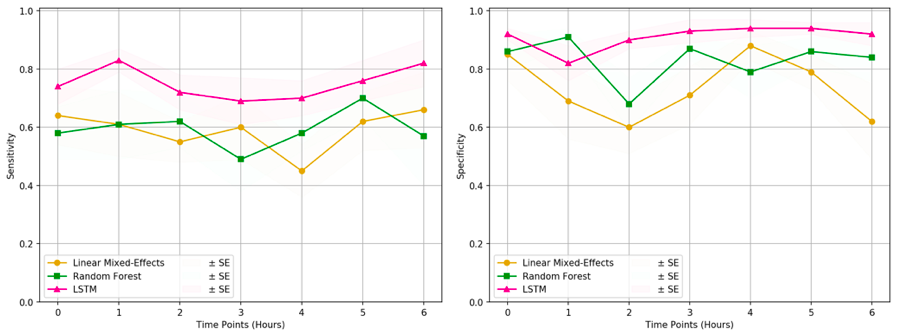
^

**C** **D**

^
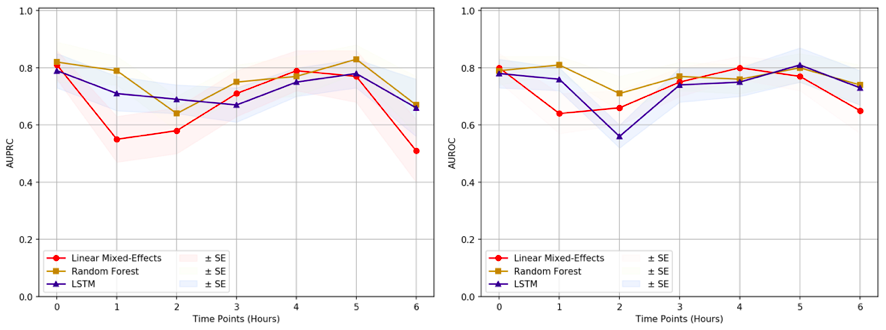
^

**E** **F**

**Supplementary Figure 1. Comparison of classification models.** The three models were compared based on their ability to classify the sequence data. It can be observed that the LSTM model outperforms the other two with respect to the **(A)** Positive Predictive Value, **(B)** Negative Predictive Value, **(C)** Sensitivity and **(D)** Specificity. **(E)** Area Under Precision-Recall Curve (AUPRC) and **(F)** Area Under Receiver Operating Characteristics (AUROC) of the models are comparable. Since our research data exhibits a high data imbalance, F1 score and AUPRC metrics are the primary considerations. LSTM model displays surpassing F1 score along with PPV, NPV, sensitivity, and specificity, making it the prime choice for this study.

^
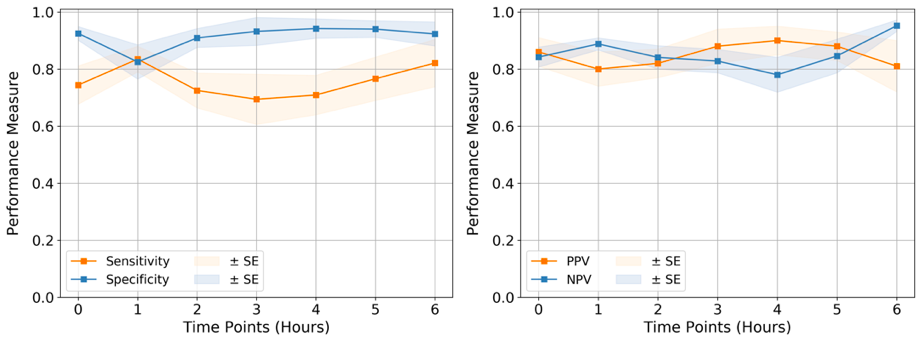
^

**A** **B**

**Supplementary Figure 2.**  **Quality Assessment:** Comparison of **(A)** Sensitivity and Specificity and **(B)** Positive Predicted Value (PPV) and Negative Predicted Value (NPV) by LSTM model at each time point till 6 hours.

**Shock detection at 0 hour using LSTM**

The CPD was extracted from every uncovered window possible using the segmented out abdomen and feet regions in a continuous way with videos sampled at 1fps. Keeping in consideration the Unique Health IDs of patients and then performing SMOTE upsampling, the observation windows of 256s (i.e. 4.26 min) were passed into LSTM networks, along with heart rate as an additional covariate, for sequence classification. 10-fold stratified cross-validation gave a mean AUPRC of 0.796 and mean AUROC of 0.788.

**Supplementary Table S2.** Comparison between the results of machine learning techniques for hemodynamic shock prediction by Nagori A. et al. and our deep learning methods, although on different sample sizes, are shown in the below table. The metrics not shown in the below table weren’t available for Nagori A. et al and hence the results cannot be compared for them. It can be observed that our deep learning techniques, along with being end-to-end without the need for handcrafted features, show an improvement in each metric by ~10%, which proves the efficacy of our method. Only a slight improvement in AUROC can be because of the imbalanced data in our case. (ML - Machine Learning, DL - Deep Learning, AUROC - Area Under Receiver Operating Characteristics, PPV - Positive predictive value, NPV - Negative predictive value, D - Detection, P - Prediction. All values are in Mean(SE))

| Time Pt. | AUROC | | Accuracy | | Sensitivity | | Specificity | | PPV | | NPV | |
| --- | --- | --- | --- | --- | --- | --- | --- | --- | --- | --- | --- | --- |
|  | DL | ML | DL | ML | DL | ML | DL | ML | DL | ML | DL | ML |
| 0hr (D) | **0.78 (0.05)** | 0.75 (0.03) | **0.85 (0.03)** | 0.73 (0.03) | **0.74 (0.06)** | 0.58 (0.06) | **0.92 (0.02)** | 0.81 (0.06) | **0.86 (0.05)** | 0.74 (0.05) | **0.84 (0.03)** | 0.75 (0.03) |
| 3hr (P) | 0.74 (0.06) | 0.77 (0.04) | **0.83 (0.04)** | 0.73 (0.03) | **0.69 (0.08)** | 0.65 (0.06) | **0.93 (0.04)** | 0.82 (0.03) | **0.88 (0.06)** | 0.75 (0.04) | **0.82 (0.04)** | 0.74 (0.05) |
| 6hr (P) | **0.73 (0.06)** | 0.68 (0.03) | **0.89 (0.03)** | 0.69 (0.02) | **0.82 (0.08)** | 0.58 (0.07) | **0.92 (0.04)** | 0.74 (0.06) | **0.81 (0.09)** | 0.79 (0.04) | **0.95 (0.02)** | 0.64 (0.02) |

**Supplementary Table S3.** The metrics observed for direct classification of images for shock detection. (SGD - Stochastic Gradient Descent, AUROC - Area Under Receiver Operating Characteristics)

| Model | Optimizer | AUROC | Accuracy |
| --- | --- | --- | --- |
| ResNet-50 | SGD | 0.60 | 65% |

**Supplementary Table S4.** Statistical significance analysis using the Hanley and McNeil formula (Hanley and McNeil, 1983). It is specifically used as our models are stratified k-fold cross-validated, and the train and test sets would be independent within each fold, but might not be across the folds. Stratified cross-validation violates the IID condition and hence Wilcoxon/student’s t-test cannot be used. Hanley, et. al. also state that single-tailed tests can be used in such cases, and a value near to the cutoff p-value can be considered as evidence that this observation may not be completely random. Hence, single-tailed analysis was performed with an alpha cutoff value of 0.10. It can be observed that AUPRC, our primary metric in use for data imbalance conditions, displays its statistical significance for most of the time points for the time window under our consideration.

| Time Pt. | AUPRC | | AUROC | |
| --- | --- | --- | --- | --- |
|  | *P*-value | Significant? | *P*-value | Significant? |
| 0hr | 0.116102 | ~Yes | 0.406949 | No |
| 1hr | 0.174508 | ~Yes | 0.169814 | ~Yes |
| 2hr | 0.002146 | Yes | 0.006782 | Yes |
| 3hr | 0.407027 | No | 0.418395 | No |
| 4hr | 0.077194 | Yes | 0.412780 | No |
| 5hr | 0.215846 | No | 0.144732 | ~Yes |
| 6hr | 0.000095 | Yes | 0.014258 | Yes |

**Supplementary Table S5.** Performance of the proposed model predicting the presence of Shock/Non-shock using automated CPD, heart rate and respiratory rate. The Time Pt. column depicts the subsequent hours from the time of taking the observation, at which the results were recorded. The unequal number of shock and non-shock sequences is due to the absence of patient data with the increasing number of hours. (S/NS - Number of Shock/Non-Shock sequences present, AUPRC - Area Under Precision-Recall Curve, AUROC - Area Under Receiver Operating Characteristics, PPV - Positive predictive value, NPV - Negative predictive value, D - Detection, P - Prediction)

| Time Pt. | S/NS | AUPRC | AUROC | Accuracy | Sensitivity | Specificity | PPV | NPV | Youden |
| --- | --- | --- | --- | --- | --- | --- | --- | --- | --- |
| 0hr | 120, 220 | 0.7 (0.04) | 0.7 (0.06) | 0.77 (0.05) | 0.56 (0.08) | 0.96 (0.02) | 0.92 (0.03) | 0.72 (0.05) | 0.61 |
| 1hr | 116, 215 | 0.75 (0.05) | 0.77 (0.05) | 0.84 (0.03) | 0.70 (0.07) | 0.92 (0.05) | 0.91 (0.04) | 0.83 (0.04) | 0.47 |
| 2hr | 120, 206 | 0.44 (0.07) | 0.61 (0.06) | 0.80 (0.04) | 0.66 (0.05) | 0.90 (0.07) | 0.83 (0.08) | 0.82 (0.03) | 0.55 |
| 3hr | 121, 190 | 0.84 (0.05) | 0.81 (0.05) | 0.88 (0.03) | 0.75 (0.08) | 0.97 (0.01) | 0.95 (0.02) | 0.86 (0.03) | 0.54 |
| 4hr | 125, 184 | 0.69 (0.03) | 0.68 (0.04) | 0.86 (0.03) | 0.72 (0.05) | 0.99 (0.01) | 0.98 (0.02) | 0.79 (0.05) | 0.5 |
| 5hr | 113, 198 | 0.81 (0.03) | 0.88 (0.03) | 0.93 (0.02) | 0.90 (0.04) | 0.95 (0.02) | 0.94 (0.02) | 0.94 (0.02) | 0.4 |
| 6hr | 111, 197 | 0.82 (0.09) | 0.87 (0.07) | 0.85 (0.07) | 0.86 (0.06) | 0.85 (0.09) | 0.82 (0.10) | 0.95 (0.02) | 0.31 |

**Additional References**

1. Kursa, M & Rudnicki, W. (2010). Feature Selection with Boruta Package. Journal of Statistical Software 36, 1-13.
2. Hanley, J. A. and McNeil, B. J. (1983). A method of comparing the areas under receiver operating characteristic curves derived from the same cases. *Radiology* 148, 839–843.
3. Dietterich, T. G. (1998). Approximate statistical tests for comparing supervised classification learning algorithms. *Neural Computation* 10, 1895–1923.
4. Bradski, G. (2000). The OpenCV Library. *Dr. Dobb’s Journal of Software Tools* 3.
5. Abadi, M. et al. (2015). TensorFlow: Large-scale machine learning on heterogeneous systems. *CoRR*.
6. Pedregosa, F. et al. (2011). Scikit-learn: Machine learning in Python. *Journal of Machine Learning Research* 12, 2825–2830.
7. Kovac, Z. and Belina, D. (1998). The pathophysiology of hemodynamic shock syndrome (part one). *Lijec Vjesn* 120, 379–392.
8. Forman, G. and Scholz, M. (2010). Apples-to-Apples in Cross-Validation Studies: Pitfalls in Classifier Performance Measurement. *Association for Computing Machinery* 12, 49–57.
